# Graphislactone A, a Fungal Antioxidant Metabolite, Reduces Lipogenesis and Protects against Diet-Induced Hepatic Steatosis in Mice

**DOI:** 10.3390/ijms25021096

**Published:** 2024-01-16

**Authors:** Yeonmi Lee, Hye-Rim Jang, Dongjin Lee, Jongjun Lee, Hae-Rim Jung, Sung-Yup Cho, Hui-Young Lee

**Affiliations:** 1 Laboratory of Mitochondria and Metabolic Diseases, Lee Gil Ya Cancer and Diabetes Institute, Gachon University, Incheon 21999, Republic of Korea; 2 Department of Health Sciences and Technology, Gachon Advanced Institute for Health Sciences and Technology (GAIHST), Gachon University, Incheon 21999, Republic of Korea; 3 Genomic Medicine Institute, Medical Research Center, Seoul National University, Seoul 03080, Republic of Koreacsybio@snu.ac.kr (S.-Y.C.); 4 Department of Biomedical Sciences, College of Medicine, Seoul National University, Seoul 03080, Republic of Korea; 5 Division of Molecular Medicine, Department of Medicine, College of Medicine, Gachon University, Incheon 21936, Republic of Korea

**Keywords:** graphislactone A, natural antioxidant, mice, high-fat diet, lipogenesis

## Abstract

Graphislactone A (GPA), a secondary metabolite derived from a mycobiont found in the lichens of the genus Graphis, exhibits antioxidant properties. However, the potential biological functions and therapeutic applications of GPA at the cellular and animal levels have not yet been investigated. In the present study, we explored the therapeutic potential of GPA in mitigating non-alcoholic fatty liver disease (NAFLD) and its underlying mechanisms through a series of experiments using various cell lines and animal models. GPA demonstrated antioxidant capacity on a par with that of vitamin C in cultured hepatocytes and reduced the inflammatory response induced by lipopolysaccharide in primary macrophages. However, in animal studies using an NAFLD mouse model, GPA had a milder impact on liver inflammation while markedly attenuating hepatic steatosis. This effect was confirmed in an animal model of early fatty liver disease without inflammation. Mechanistically, GPA inhibited lipogenesis rather than fat oxidation in cultured hepatocytes. Similarly, RNA sequencing data revealed intriguing associations between GPA and the adipogenic pathways during adipocyte differentiation. GPA effectively reduced lipid accumulation and suppressed lipogenic gene expression in AML12 hepatocytes and 3T3-L1 adipocytes. In summary, our study demonstrates the potential application of GPA to protect against hepatic steatosis in vivo and suggests a novel role for GPA as an underlying mechanism in lipogenesis, paving the way for future exploration of its therapeutic potential.

## 1. Introduction

Metabolic diseases, such as obesity, type 2 diabetes, and cardiovascular diseases, are growing globally [1,2]. Non-alcoholic fatty liver disease (NAFLD) is emerging as a prominent public health issue affecting approximately 25% of the global population [3]. NAFLD is a broad term that encompasses all the non-alcohol-related fatty liver conditions, ranging from simple steatosis to non-alcoholic steatohepatitis (NASH), both of which are important for the prevention and treatment of related metabolic diseases. NAFLD progression is caused by several factors and is referred to as the “multiple hit” hypothesis [4,5]. Simple steatosis is characterized by the excessive accumulation of triglyceride (TG) within hepatocytes, and it is referred to as the initial hit in the “multiple hit” hypothesis in the NAFLD pathogenesis [5]. The primary abnormality (simple steatosis) is most likely attributed to excessive lipid intake and insulin resistance, a condition closely linked to obesity and insulin resistance [6,7], which leads to lipid accumulation in the liver. The accumulated hepatic lipids sensitize the liver to injury from a variety of causes, leading to more advanced stages of NAFLD, known as NASH [7]. Possible candidates for multiple hits include increased oxidative stress, lipid peroxidation, and the generation of gut-derived toxins, such as lipopolysaccharide (LPS) [5]. NASH is the most severe manifestation of NAFLD and is marked by inflammation and hepatocyte damage [6,7], which not only leads to cardiovascular disease and type 2 diabetes but also substantially increases the risk of hepatic complications such as cirrhosis and hepatocellular carcinoma [8]. 

Oxidative stress is characterized by an imbalance between the production of reactive oxygen species (ROS) and the antioxidant defense systems. Overproduction of ROS can cause various metabolic diseases, including obesity, insulin resistance, and chronic inflammation. It has been one of the most popular proposed mechanisms of hepatocellular injury and is central to the pathogenesis of NAFLD [5,9,10,11]. ROS-induced lipid peroxidation leads to hepatocellular damage and inflammation, which have been implicated in NASH progression [12] via contributing to the target organ damage [13]. Antioxidants have the potential to disrupt the cascade of events that lead to NAFLD progression, as demonstrated by various in vivo studies. Clinical studies have affirmed their efficacy in disrupting NASH; for instance, 11 obese children with NASH experienced normalized aminotransferase levels after 4–10 months of daily vitamin E administration (400–1200 IU) [14]. Another study involving adults treated with vitamin E (300 mg/d) for 1 year showcased improved steatosis and decreased transforming growth factor beta 1 (TGF-β1) levels [15]. Vitamin C intake protects against NAFLD in both men and non-obese patients [16]. In rodent models, dietary vitamin E intake protects against oxidative stress-related liver injury [17] and prevents sterol regulatory element-binding protein-1 (SREBP-1) maturation in HepG2 cells [16], whereas other antioxidants, such as resveratrol, curcumin, quercetin, and anthocyanin, have anti-inflammatory, anti-obesity, and anti-dyslipidemic functions [18]. Therefore, investigating antioxidants and natural or synthetic compounds that can neutralize ROS and mitigate oxidative stress represents a promising avenue for NAFLD therapeutics.

Graphislactone A (GPA) is the major analogue of a family of phenolic benzopyranones isolated from a mycobiont of lichens of the genus Graphis, first reported by Japanese researchers at Kobe Pharmaceutical University in 1997 [19]. In 2005, GPA was screened from the fungus *Cephalosporium* sp. IFB-E001 and characterized as the most bioactive secondary metabolite of the fungus with its free radical scavenging and antioxidant activities in in vitro experiments found to be greater than those of strong antioxidants, such as butylated hydroxytoluene and ascorbic acid [20]. Since then, despite the robust antioxidant properties of GPA in vitro, the potential biological functions and therapeutic applications of GPA at the cellular and animal levels have not been explored. In the present study, we first investigate whether GPA can regulate inflammation and lipid metabolism, and then if it can protect against NAFLD, by conducting a series of experiments using various cell lines and animal models.

## 2. Results

### 2.1. GPA Reduces H_2_O_2_-Induced ROS Production in AML12 Hepatocytes and LPS-Induced Inflammatory Gene Expression in Primary Macrophages

**Figure 1:**
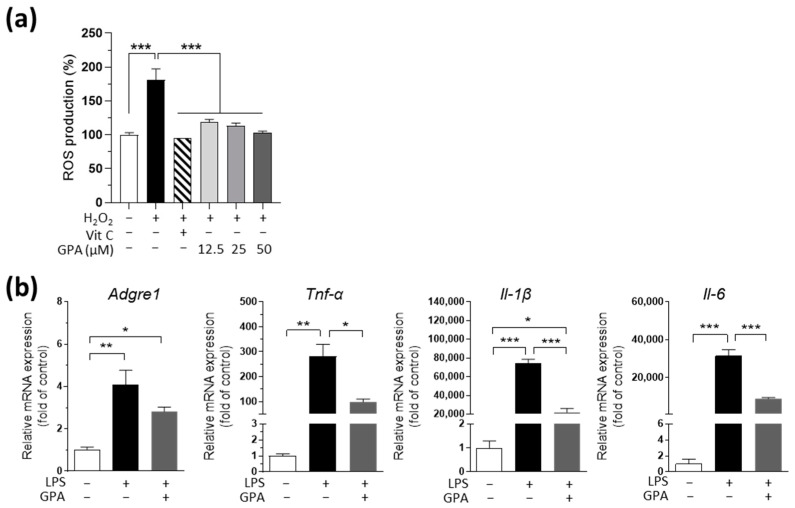
GPA reduces ROS production in AML12 hepatocytes and the inflammatory response in primary macrophages. (**a**) The cellular ROS production under an H_2_O_2_-stimulated condition after various doses of GPA treatment in AML12 cells. (**b**) The mRNA expression of LPS-stimulated inflammatory genes in primary peritoneal macrophages after 50 μM GPA treatment. All the experiments were performed in triplicate, and data are expressed as the mean ± the mean of the standard error. Asterisks (* *p* < 0.05, ** *p* < 0.01, and *** *p* < 0.001) indicate statistically significant differences via one-way ANOVA with Bonferroni’s post hoc test. H_2_O_2_, hydrogen peroxide; Vit C, vitamin C; GPA, graphislactone A; *Adgre1*, adhesion G protein-coupled receptor E1; *Tnf-α*, tumor necrosis factor-alpha; *Il-1β*, interleukin-1beta; *Il-6*, interleukin-6; LPS, lipopolysaccharide.

We first tested whether GPA reduced cellular ROS production as an antioxidant, as previously suggested [20]. GPA was treated in AML12 hepatocytes at concentrations of 12.5 μM, 25 μM, and 50 μM. These concentrations were selected based on GPA’s non-cytotoxic data at levels below 125 μM, maintaining cell viability above 80%. GPA showed ROS-scavenging effects on H_2_O_2_-induced ROS production, similar to those of vitamin C in AML12 hepatocytes (Figure 1a). As gut-derived toxins, such as LPS, are known to induce oxidative stress and inflammation in the liver [21] and are implicated as a second hit in the pathogenesis of NAFLD [5], we further tested LPS-stimulated macrophages to determine whether GPA has anti-inflammatory effects. In primary peritoneal macrophages, there was a pronounced increase in the gene expression of pro-inflammatory cytokines, such as tumor necrosis factor-alpha (*Tnf-α*), interleukin-6 (*Il-6*) and interleukin-1 beta (*Il-1β*), along with the adhesion G protein-coupled receptor E1 (*Adgre1*) gene, encoding a unique marker of murine macrophages, F4/80 (Figure 1b). GPA significantly decreased the expression of these cytokines (Figure 1b). The anti-inflammatory response of GPA was verified using a different cell line, RAW 264.7, a murine macrophage cell line, with results similar to those in primary macrophages. GPA reduced the genes of pro-inflammatory cytokines such as *Tnf-α*, *Il-6*, and *Il-1β* but not *Adgre1* (Supplementary Figure S1). 

### 2.2. GPA Attenuates Steatosis but Not Inflammation in the Liver of Choline-Deficient, L-amino Acid-Defined, High-Fat Diet (HFD)-Induced NASH Mice

**Figure 2:**
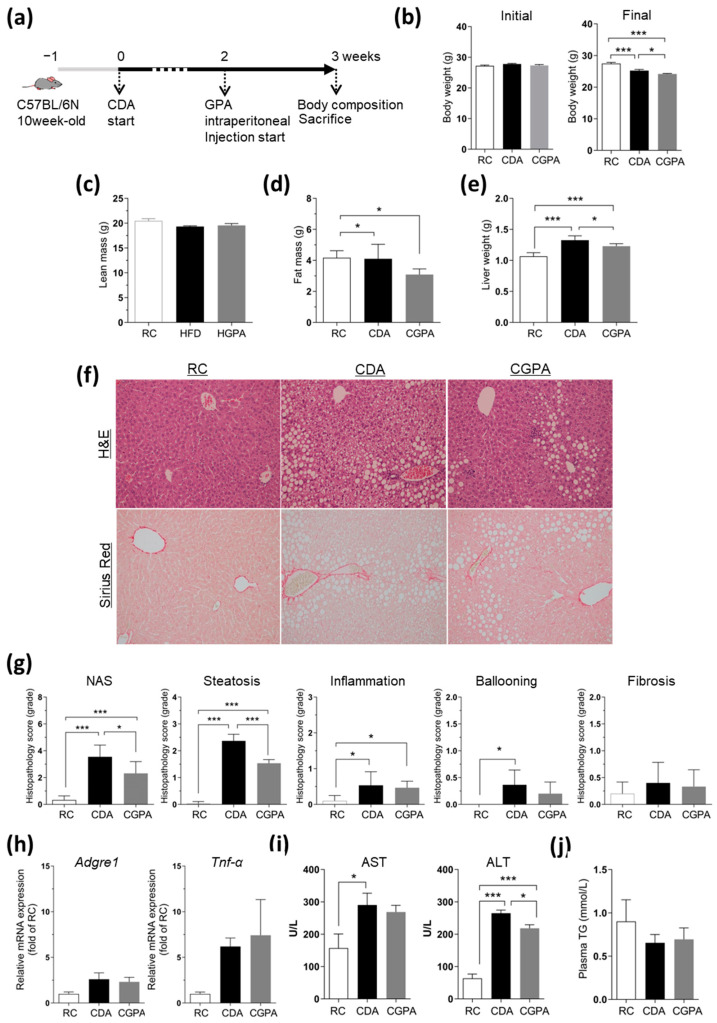
GPA attenuates steatosis but not inflammation in the liver of CDA-induced NASH mice. (**a**) A schematic diagram of the experiment schedule using a NASH mice model. Mice were fed CDA for three weeks, with GPA administered daily through intraperitoneal route during the final week. (**b**) Initial and final body weight of all the experimental groups, including regular chow-fed control mice (RC), CDA-fed (CDA), and CDA-fed mice with GPA (CGPA). (**c**) Lean body mass and (**d**) fat mass evaluated via ^1^H-NMR system. (**e**) Liver was weighted and (**f**) slide section was stained by H&E (upper) and Sirius red (lower) staining, and (**g**) evaluated for NAS, including steatosis, inflammation, ballooning, and fibrosis. (**h**) The hepatic mRNA expression of inflammatory genes, (**i**) plasma AST and ALT levels and (**j**) TG concentration in the RC, CDA and CGPA groups. *n* = 4–6 for each group. The data are expressed as the mean ± the mean of the standard error. Asterisks (* *p* < 0.05 and *** *p* < 0.001) indicate statistically significant differences using a one-way ANOVA with Bonferroni’s post hoc analysis. NASH, non-alcoholic steatohepatitis; CDA, choline-deficient, L-amino acid-defined, HFD, high-fat diet; GPA, graphislactone A; RC, regular chow; H&E, hematoxylin and eosin; NAS, non-alcoholic fatty liver disease activity score; *Adgre1*, adhesion G protein-coupled receptor E1; *Tnf-α*, tumor necrosis factor-alpha; AST, aspartate transaminase; ALT, alanine transaminase; TG, triglyceride.

Next, we tested whether the antioxidant and anti-inflammatory properties of GPA observed in our cell studies were evident in animal models of NASH. We fed mice a choline-deficient, L-amino acid-defined, HFD (CDA), known to induce liver steatosis and inflammation within 3-4 weeks, representing a moderate NASH model [22,23]. Following a one-week acclimation period, the mice were maintained on CDA for 3 weeks, with GPA administered daily through the intraperitoneal route during the final week (Figure 2a). GPA was injected at a concentration of 10 mg per mouse body weight because GPA’s effective concentration in in vitro experiments was below 50 μM (15.114 mg/kg). After 3 weeks of CDA feeding, there was a decrease in body weight (Figure 2b) and lean body mass (Figure 2c) in the CDA-fed group compared with the regular chow (RC)-fed group. Throughout the CDA feeding, the body fat mass was slightly reduced in the GPA-injected group compared to the group without GPA (Figure 2d), whereas the lean body mass remained unchanged by the GPA treatment, as shown in the CDA-fed group with GPA (CGPA) (Figure 2b,c). Despite the absence of an increase in body weight, there was an increase in the liver weight during CDA feeding, which was significantly reduced in the CGPA group (Figure 2e). The increased liver weight appears to be attributable to excessive fat deposition, as indicated by histopathological findings that showed extensive infiltration of lipid vacuoles in the livers of CDA-fed mice compared to that in RC-fed livers (Figure 2f). Characteristically, zone 1 around the portal triad showed a pattern of macro-fat vacuole infiltration, whereas zones 2 and 3 around the central vein showed a pattern of micro-fat vacuole infiltration (Figure 2f). All of these were reduced by GPA treatment, leading to a decreased NAFLD activity score (NAS) in the GPA-treated livers compared to that in the non-treated CDA livers (Figure 2g). Upon GPA treatment, there were no differences in fibrosis, as evaluated using Sirius red staining (Figure 2f), or liver injury, as indicated by inflammation, ballooning, and fibrosis via histopathological scoring (Figure 2g). Similarly, other markers of liver injury, including hepatic *Adgre1*, *Tnf-α* mRNA expression, and plasma aspartate transaminase (AST) and alanine transaminase (ALT) concentrations, exhibited a significant increase in the CDA-fed mice compared to the RC-fed mice (Figure 2h,i). However, no significant reduction was observed as a result of GPA treatment in any of these, except for the ALT levels (Figure 2i). The plasma TG levels were slightly lower in the CDA-fed mice than in the RC-fed mice (Figure 2j). These data indicate that GPA likely affects lipid accumulation, rather than inflammation, during the initial stages of NAFLD progression in the animal model.

### 2.3. GPA Attenuates Steatosis and Adiposity in HFD-Induced Obesity Mice

**Figure 3:**
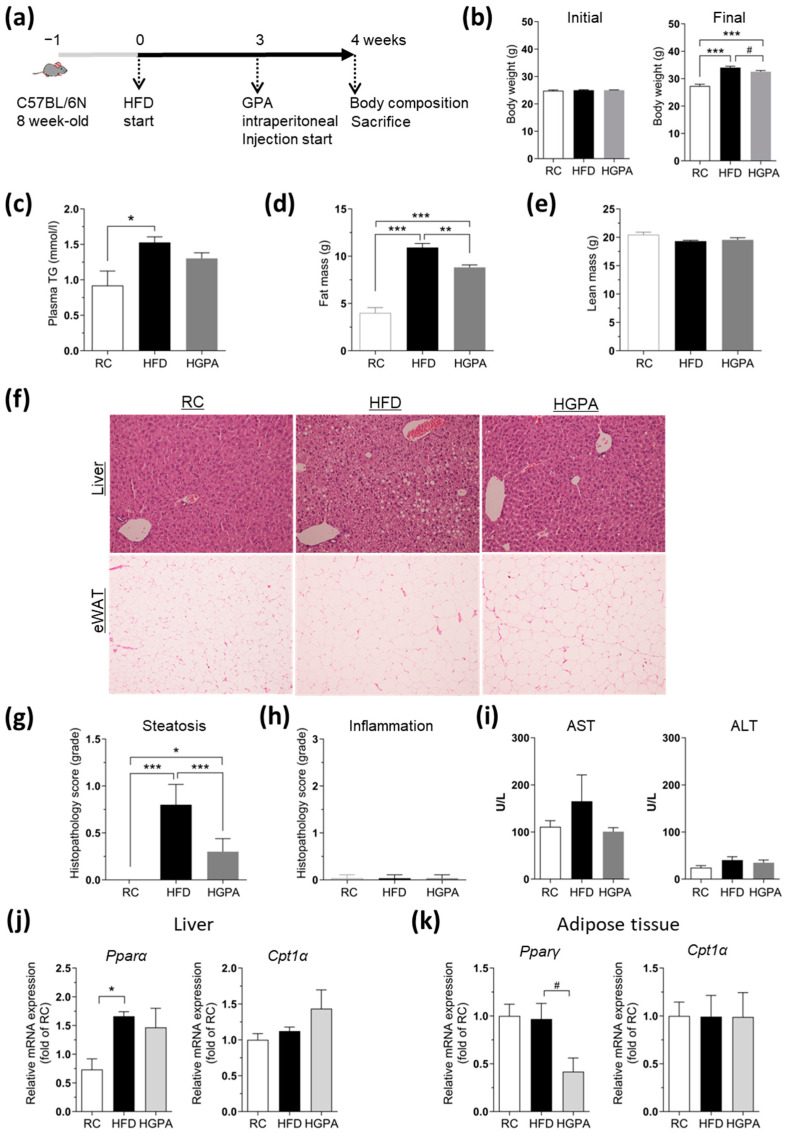
GPA attenuates steatosis and adiposity in HFD-induced obesity mice. (**a**) A schematic diagram of the in vivo study using HFD-fed mice. Mice were fed an HFD for 4 weeks, with GPA administered daily through intraperitoneal route during the final week. (**b**) The initial body weight and final body weight of all the experimental groups, including regular chow-fed control mice (RC), HFD-fed (HFD), and HFD-fed mice with GPA (HGPA). (**c**) The plasma TG concentration in the RC, HFD and HGPA groups. (**d**) The lean body mass and (**e**) the fat mass analyzed via the ^1^H-NMR system. (**f**) H&E staining and (**g**) the steatosis and (**h**) inflammation scoring for the liver of each group. (**i**) The hepatic mRNA expression and (**k**) the adipose mRNA expression of lipid metabolism-related genes in the RC, HFD and HGPA groups. The plasma AST and ALT levels in the RC, HFD and HGPA groups. (**j**) *n* = 4–9 per each group. The data are expressed as the mean ± the mean of the standard error. Asterisks (* *p* < 0.05, ** *p* < 0.01, and *** *p* < 0.001) indicate statistically significant differences using a one-way ANOVA with Bonferroni’s post hoc analysis. Sharps (^#^ *p* < 0.05) indicate statistically significant differences via a two-tailed unpaired Student’s *t*-test. HFD, high-fat diet; GPA, graphislactone A; RC, regular chow; TG, triglyceride; H&E, hematoxylin and eosin; AST, aspartate transaminase; ALT, alanine transaminase; *Pparα*, peroxisome proliferator-activated receptor alpha; *Cpt1α*, carnitine palmitoyltrasferase 1 alpha.

Choline is an essential factor for the synthesis of very-low-density lipoproteins (VLDL) in the liver [24,25], and it is a significant carrier of fat in the bloodstream and tissues. Consequently, our CDA-fed mice exhibited weight loss (Figure 2b) and decreased plasma TG levels compared to the RC-fed mice (Figure 2j). Furthermore, the most notable effect of GPA treatment on liver steatosis was observed in the CDA-induced NASH model. Therefore, to further investigate the effect of GPA on lipid accumulation in the early stages of NAFLD, we fed mice an HFD. After a one-week acclimation period, the animals were administered an HFD for 4 weeks, with GPA injected daily through the intraperitoneal route during the final week (Figure 3a). In contrast to the CDA model, significant increases in body weight and plasma TG levels were observed after HFD feeding (Figure 3b,c). These increases in body weight were significantly reduced (Figure 3b), with a trend toward a nonsignificant reduction in plasma TG levels, following GPA administration (Figure 3c). The changes in body weight were mostly accounted for by changes in body fat mass and not lean body mass (Figure 3d,e). Consistent with the data from our CDA-induced NASH mice (Figure 2), GPA administration decreased liver steatosis (Figure 3f) and the steatosis scores (Figure 3g) in HFD mice. Furthermore, liver inflammation (Figure 3h) and plasma ALT/AST (Figure 3i) were not altered by either HFD feeding or GPA treatment in any group, indicating that GPA reduces hepatic lipid accumulation and body fat mass independent of its anti-inflammatory characteristics at the early stage of NAFLD. There were no significant differences in mRNA expression for either lipogenic or oxidative genes with GPA in the liver tissue (Figure 3j), but a notable decrease in the peroxisome proliferator-activated receptor gamma (*Pparγ*) mRNA expression was observed in adipose tissue with GPA administration (Figure 3k). Collectively, these data confirm that GPA plays a pivotal role in mitigating hepatic steatosis and further imply an extended role beyond the liver in lipid accumulation, independent of its anti-inflammatory character at the early stage of NAFLD.

### 2.4. GPA Reduces Lipid Accumulation and Lipogenic Gene Expression but Does Not Increase Mitochondrial Fatty Acid Oxidation in Hepatocytes

**Figure 4:**
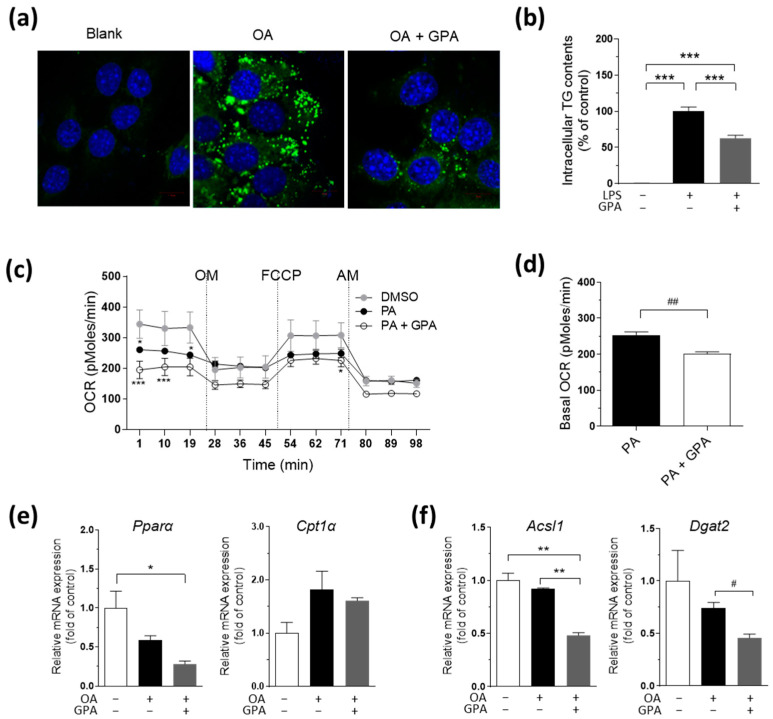
GPA reduces lipid accumulation and lipogenesis gene expression in hepatocytes. (**a**) In AML12 hepatocytes, Bodipy^TM^ staining for OA-induced lipid droplets after 50 μM GPA treatment for 18 h (green: lipid staining with Bodipy^TM^, blue: nuclei staining with DAPI). (**b**) Intracellular TG contents for OA-induced lipid droplets after various doses of GPA treatment. (**c**) In AML12 cells with and without GPA following 24 h PA treatment, the changing patterns of cellular OCR in response to mitochondrial inhibitors or stimulator. (**d**) The basal OCR of AML12 hepatocytes with and without GPA following 24 h PA treatment. (**e**) The mRNA expression of oxidative-related genes in AML12 hepatocytes after GPA treatment. (**f**) The mRNA expression of lipogenesis-related genes in AML12 hepatocytes after GPA treatment. *n* = 3 per group. The data are expressed as the mean ± the mean of the standard error. Asterisks (* *p* < 0.05, ** *p* < 0.01, and *** *p* < 0.001) indicate statistically significant differences using a one-way ANOVA with Bonferroni’s post hoc analysis. For panel (**c**), statistical analysis involves a two-way ANOVA with Bonferroni's post hoc analysis, and significant differences are indicated by asterisks in comparison to the DMSO group. Sharps (^#^ *p* < 0.05 and ^##^ *p* < 0.01) indicate statistically significant differences via a two-tailed unpaired Student’s *t*-test for the comparison of two groups. OA, oleic acid; GPA, graphislactone A; TG, triglyceride; OCR, oxygen consumption rate; PA, palmitic acid; OM, oligomycin; FCCP, carbonyl cyanide-p-trifluoromethoxyphenylhydrazone; AM, antimycin A; DMSO, dimethyl sulfoxide; *Pparα*, peroxisome proliferator-activated receptor alpha; *Cpt1α*, carnitine palmitoyltrasferase 1 alpha; *Acsl1*, acyl-CoA synthetase long-chain family member 1; *Dgat2*, diacylglycerol o-acyltransferase 2.

To further explore the effect of GPA on NAFLD progression and lipid accumulation, we investigated whether GPA alters oleic acid (OA)-induced lipid accumulation in cultured hepatocytes. After 18 h of co-cultivation in AML12 hepatocytes, OA markedly increased intracellular lipid accumulation, as indicated by green staining with Bodipy^TM^ (Figure 4a) and quantified by measuring the intracellular TG content (Figure 4b). OA-induced lipid droplet formation was dramatically inhibited by GPA treatment (Figure 4a), and the TG concentration was consistently reduced (Figure 4b). To investigate whether the reduced lipid accumulation in the hepatocytes caused by GPA was due to enhanced lipid oxidation, we measured the cellular oxygen consumption rate (OCR) using a Seahorse XF24 analyzer. After 18 h of treatment, palmitic acid (PA) and GPA exhibited a trend toward decreasing the oxygen consumption capacity of AML12 cells compared to the vehicle control (dimethyl sulfoxide [DMSO]) group (Figure 4c). Notably, basal OCR displayed a tendency to decrease following GPA treatment (Figure 4f), which should have increased if fatty acid oxidation was the underlying mechanism attributed to the reduced lipid accumulation in the hepatocytes (Figure 4a,b). The expression of *Pparα* mRNA showed a decreasing trend with GPA treatment, whereas no significant changes were observed in carnitine palmitoyltrasferase 1 alpha (*Cpt1α*) expression compared to the OA-treated cells. Extended analysis of the lipogenic genes, including acyl-CoA synthetase long-chain family member 1 (*Acsl1*) and diacylglycerol acyltransferase 2 (*Dgat2*) genes, demonstrated a significant decrease with GPA treatment (Figure 4f). Collectively, these data demonstrate that GPA inhibits lipid accumulation by regulating lipogenesis independently of fatty acid oxidation in an in vitro model of NAFLD.

### 2.5. GPA Down-Regulates Adipogenesis Gene Expression and Involves Inflammatory Gene Expression in 3T3-L1 Adipocytes

**Figure 5:**
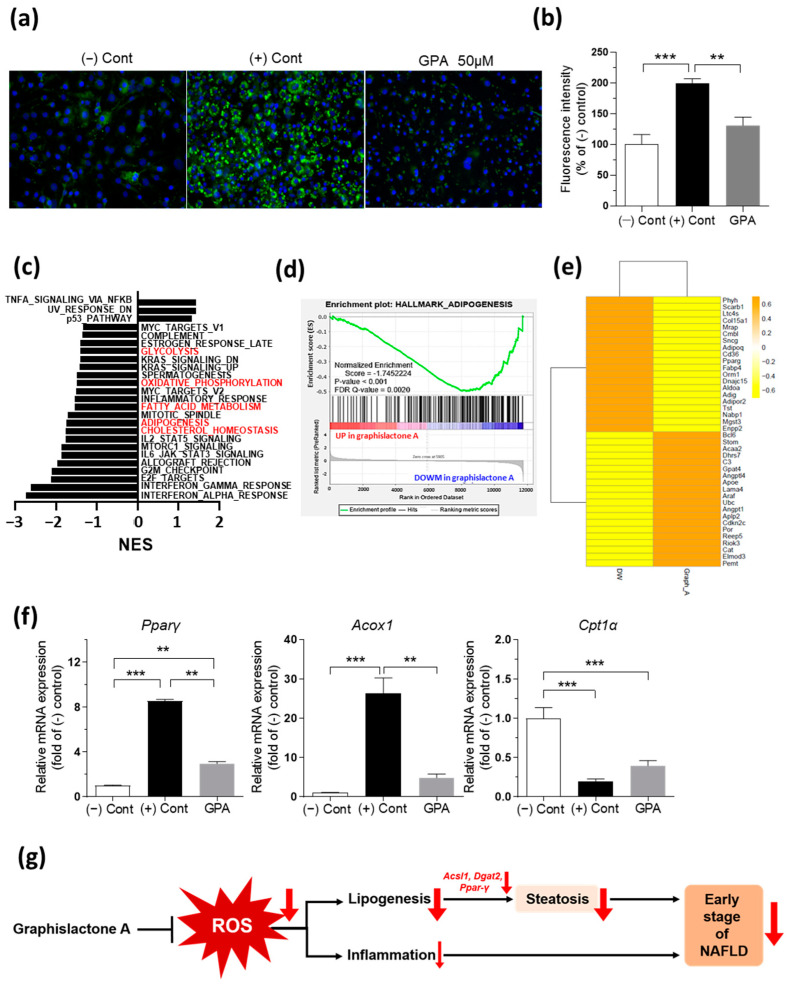
GPA reduces inflammatory and adipogenesis gene expression in 3T3-L1 adipocytes. (**a**) In 3T3-L1 adipocytes, Bodipy^TM^ staining for accumulated lipids during differentiation treated with/without 50 μM GPA for 7 d (green: lipid staining with Bodipy^TM^, blue: nuclei staining with DAPI). (**b**) The fluorescent intensity of Bodipy^TM^-stained lipid with/without 50 μM GPA for 7 d. *n* = 6 per group. (**c**) The up- and down-regulated gene sets from a GSEA with hallmark gene sets in differentiated 3T3-L1 adipocyte treated with/without GPA (*p* < 0.05). Gene sets marked in red are metabolism-related gene sets. (**d**) Enrichment plot for the adipogenesis gene set among the down-regulated gene sets in a GSEA with hallmark gene sets. (**e**) Heatmap of the top 20 up- and down-regulated genes via the treatment with GPA in the adipogenesis gene set. (**f**) The mRNA expression of lipid metabolism-related genes in 3T3-L1 adipocytes during and after 50 μM GPA treatment for 7 d. *n* = 3–9 per group. (**g**) A scheme of the proposed mechanism of GPA against the early stage of NAFLD. The data are expressed as the mean ± the mean of the standard error. Asterisks (** *p* < 0.01, and *** *p* < 0.001) indicate statistically significant differences using a one-way ANOVA with Bonferroni’s post hoc analysis. Cont, control; GPA, graphislactone A; GSEA, gene set enrichment analysis; NES, normalized enrichment score; DW, distilled water; *Pparγ*, peroxisome proliferator-activated receptor gamma; *Acox1*, peroxisomal acyl-coenzyme A oxidase 1; *Cpt1α*, carnitine palmitoyltrasferase 1 alpha.

Based on insights from both animal studies, which indicated the primary involvement of GPA in lipid accumulation (Figure 2 and Figure 3), and hepatocyte findings, which suggested a mechanism distinct from lipid oxidation (Figure 4), experiments were conducted to determine the role of GPA in adipocyte differentiation using 3T3-L1 cells. The cells were differentiated for 7 d by adding differentiation medium containing a mixture of 3-isobutyl-1-metylxanthine (IBMX), dexamethasone, and insulin, and the lipid accumulation was evaluated using Bodipy^TM^ staining. Consistently, GPA inhibited the lipid droplets in the differentiated 3T3-L1 cells (Figure 5a) and decreased the fluorescence intensity, which was measured by dissolved the Bodipy^TM^-stained lipids in 50 μM GPA (Figure 5b). Transcriptomic analysis using gene set enrichment analysis (GSEA) was performed by comparing vehicle- and GPA-treated 3T3-L1 cells. GSEA with hallmark gene sets identified three up-regulated pathways and 22 down-regulated pathways in GPA-treated adipocytes (*p* < 0.05; Figure 5c, Supplementary Tables S1 and S2). As consistent with the hepatocyte and animal experiments, several pathways related to metabolism were identified in the down-regulated pathways, such as “glycolysis”, “oxidative phosphorylation”, “fatty acid metabolism”, “adipogenesis” and “cholesterol homeostasis”, which can effect adipocyte differentiation in GPA-treated adipocytes (Figure 5c). Notably, the “Adipogenesis” pathway showed a strong correlation in plot (Figure 5d), and *Pparγ* and a cluster of differentiation 36 (*Cd36*) genes were included in the top 20 down-regulated genes (Figure 5e, Supplementary Table S3). Moreover, the mRNA expression results showed significant reductions in the expression of the *Pparγ* gene in GPA-treated adipocytes, independent of genes related to fatty acid oxidation, such as peroxisomal acyl-coenzyme A oxidase (*Acox1*) and *Cpt1a* (Figure 5f), supporting the inhibitory role of GPA in adipogenesis distinct from lipid oxidation as observed in the hepatocyte experiment (Figure 4). In terms of the inflammatory response, however, GPA treatment showed complicated results because the inflammation-related pathways were enriched in both up-regulated and down-regulated pathways (Figure 5c) as “TNF-α signaling through NF-κB” was identified in the up-regulated pathways whereas “interferon (INF)-α/γ response”, “IL-6/JAK/STAT2 signaling”, and “IL-2/STAT5 signaling” were identified in the down-regulated pathways in the GPA-treated adipocytes (Figure 5c). While individual data may introduce some complexities, considering the established role of inflammatory cytokines in adipose tissue in NAFLD development [26], this suggests a potential role of GPA in lipid accumulation beyond adipose tissue. At least, the decrease in “INF-α/γ response” could align with a clinical study indicating that serum INF-α2 was increased in obese NAFLD subjects, with a positive correlation to intramuscular TG accumulation [27].

## 3. Discussion

In 2005, GPA was first screened from the fungus *Cephalosporium* sp. IFB-E001 and characterized as the most bioactive secondary metabolite of the fungus, with its antioxidant activities found to be greater than those of ascorbic acid [20]. The antioxidant function of GPA reported earlier was not validated by directly assessing its impact on cells. Instead, it was a test tube-level experiment to determine its physicochemical antioxidant properties and scavenging potential [20]; thus, the biological role of GPA as an antioxidant has not been demonstrated for decades. To the best of our knowledge, this study is the first to confirm that GPA exhibits antioxidant functions at the cellular level. Furthermore, our data also support the idea that the antioxidant capacity of GPA plays a crucial role in lipid metabolism at both the cellular and animal levels. First, we found that GPA inhibited not only the pro-inflammatory response in cultured macrophages but also lipid accumulation in cultured hepatocytes and adipocytes. Second, GPA affected lipid accumulation rather than inflammation during the initial stages of NAFLD progression in both animal models. Finally, GPA reduced lipid accumulation through a mechanism that reduced adipogenesis but was distinct from lipid oxidation. 

In the present study, we focused solely on early-stage NAFLD and the direct mechanisms that regulate lipogenesis. The pathological role of ROS in NAFLD mostly studied the lipotoxicity and inflammation that are implicated in the target organ damage [5,9,10,11,15,17]. However, ROS may also play a major role below the damage level, even in benign conditions of NAFLD such as simple steatosis, which is characterized by lipid accumulation with less inflammation and damage. Although it is not known exactly how ROS regulate cellular lipid metabolism other than by regulating the inflammatory pathways, it is known that ROS can regulate the lipogenesis pathway both directly and indirectly. Several studies have demonstrated that ROS can directly modulate lipogenesis at the signaling level. ROS such as hydrogen peroxide are known to activate signaling pathways that promote lipogenic gene expression [28]. Vitamin E inhibits lipogenesis by preventing the maturation of the *SREBP-1* in HepG2 cells [16]. Consistent with these findings, in our study, GPA reduced lipid accumulation in the liver and adipose tissue through a mechanism linked with lipogenesis. In hepatocytes, GPA significantly decreased the expression of lipogenic genes, including *Acsl1* and *Dgat2*, that are proposed for fatty acid esterification into TG in hepatocytes [29] and animals [30]. Reduced lipid accumulation in hepatocytes caused by GPA is independent of an enhanced lipid oxidation mechanism, as demonstrated by measuring the cellular oxygen consumption rate. Similarly, the mRNA expression of *Pparγ*, a key lipogenic transcription factor, was notably decreased by the GPA treatment in both 3T3-L1 adipocytes and adipose tissue from HFD-fed mice. 

ROS can also indirectly modulate lipogenesis by influencing ROS target proteins related to insulin signaling [31,32,33] and mitochondrial dysfunction [34,35], which can effect cellular lipogenesis and incomplete fatty acid oxidation, respectively. ROS-susceptible proteins are an example of an indirect effect of ROS on the regulation of lipid metabolism. The phosphatase and tensin homolog deleted on chromosome 10 (PTEN) is a major target of ROS because of the oxidation-susceptible nucleophilic cysteine at its active site. PTEN inhibits the phosphatidylinositol-3-kinase (PI3K)/AKT pathway of insulin signaling, and the oxidation of PTEN by ROS enhances insulin sensitivity [36], which induces lipogenic processes. Hepatocyte-specific PTEN deficiency induced massive hepatomegaly and TG accumulation [32,37]. Putting aside the debate on the suitability of employing PTEN regulation to control fat metabolism or NASH, this is undeniably a compelling example demonstrating the impact of ROS on the regulation of lipid metabolism. Because of the robust antioxidant capacity of GPA, it is worth conducting long-term follow-up studies to fully investigate the ROS target proteins or mitochondrial function, which will expand our understanding of the role of GPA in glucose and lipid metabolism, linking ROS scavenging to insulin resistance and chronic metabolic diseases.

While GPA exhibited consistent effectiveness in regulating lipogenesis in this study, its anti-inflammatory role appears to require a more complex interpretation. GPA shows an anti-inflammatory function in macrophages following LPS stimulation, but conflicting outcomes have presented within adipocytes. While the TNFα pathway exhibited an up-regulation in GPA-treated adipocytes, the overall inflammatory response pathways showed a down-regulation. Moreover, animal studies have indicated that the anti-inflammatory effect of GPA is milder than its effect on lipogenesis. This discrepancy may stem from the assessment in the early stage of NAFLD or adipocyte differentiation, which are relatively mild inflammatory conditions. Nevertheless, considering the robust antioxidant function of GPA in macrophages, its potential role in environments with pronounced inflammatory responses, such as LPS or H_2_O_2_ stimulation, is apparent. To explore this further, it is necessary to utilize an animal model representing late-stage NASH induced over an extended period or to evaluate its effects using alternative inflammatory disease models.

In conclusion, this study is the first to reveal the pathophysiological roles of GPA in hepatic steatosis at the cellular and animal levels, suggesting the potential therapeutic applications of GPA in NAFLD and obesity-related metabolic diseases.

## 4. Materials and Methods

### 4.1. H_2_O_2_-Stimualted ROS Measurement in Hepatocytes

The H_2_O_2_-stimualted ROS production was measured in AML12 hepatocytes. AML12, a murine hepatocyte cell line (American Type Culture Collection; ATCC, Manassas, VA, USA), was cultured at 37 °C under 5% CO_2_ in Dulbecco’s modified Eagle’s medium (DMEM)/Ham’s F12 medium (Welgene, Daegu, Republic of Korea) supplemented with 10% (*v*/*v*) heat-inactivated fetal bovine serum (FBS), 1% insulin-transferrin-selenium (ITS) (Thermo Fishers Scientific, Waltham, MA, USA), 40 ng/mL dexamethasone (Sigma-Aldrich, St. Louis, MO, USA), 100 U/mL of penicillin, and 100 μg/mL streptomycin. Graphislactone A (GPA) was purchased from BioAustralis (Smithfield, Australia) for the experiments. The hepatocytes, at a density of 1 x 10^4^ cells/well in a 24-well plate, were treated with 500 μM H_2_O_2_ (Sigma-Aldrich, St. Louis, MO, USA) for 10 min after adding GPA for 1 h. The medium was removed and washed with PBS (Welgene, Daegu, Republic of Korea). ROS production in AML12 cells was measured at 560 nm via an Amlex^TM^ Red Hydrogen Peroxide/Peroxidase Assay Kit (Thermo Fishers Scientific, Waltham, MA, USA) as per the manufacturer’s instruction.

### 4.2. LPS-Induced Inflammatory Response in Macrophages

The LPS-induced inflammatory response was evaluated in peritoneal macrophages isolated from mice and the Raw264.7 cell line. Peritoneal macrophages from 10- to 12-week-old male C57BL/6N mice (Orient Bio, Seongnam, Republic of Korea) were harvested via peritoneal lavage with 10 mL of ice-cold PBS 4 d after intraperitoneal injection of 4% thioglycolate (1 mL) (Sigma-Aldrich, St. Louis, MO, USA). The retrieved fluid was centrifuged at 400× *g* for 10 min at 4 °C, and the cell pellet was re-suspended in RPMI-1640 medium (Welgene, Daegu, Republic of Korea) supplemented with 100 U/mL of penicillin, 100 μg/mL streptomycin, and 10% (*v*/*v*) heat-inactivated FBS (Welgene, Daegu, Republic of Korea). Fresh isolated primary peritoneal macrophage was cultured at 37 °C under 5% CO_2_ in RPMI-1640 medium with 100 U/mL of penicillin, 100 μg/mL streptomycin and 10% (*v*/*v*) heat-inactivated FBS. Raw 264.7, a murine macrophage cell line, was purchased from the ATCC (Manassas, VA, USA) and cultured in DMEM (Welgene, Daegu, Republic of Korea) with 100 U/mL of penicillin, 100 μg/mL streptomycin and 10% (*v*/*v*) heat-inactivated FBS. GPA was purchased from BioAustralis (Smith Field, Australia).

The macrophages, at a density of 2 × 10^4^ of cells per well in a 12-well plate, were exposed to 10 nM LPS (Sigma-Aldrich, St. Louis, MO, USA) with/without 50, 25, or 12.5 μM GPA for 6 h. Treated cells were collected for real-time PCR analysis.

### 4.3. Fatty Acid-Induced Lipid Accumulation in Hepatocytes

The AML12 hepatocytes, at a density of 2 × 10^4^ cells per well in a 12-well plate, were co-treated with 200 μM bovine serum albumin (BSA)-conjugated OA (Sigma-Aldrich, St. Louis, MO, USA) and/or GPA for 18 h. The TG content was measured using a TG Quantification Assay Kit (Abcam, Cambridge, UK) according to the manufacturer’s instructions. For cell imaging purposes, the lipid droplets were stained with Bodipy^TM^ 493/503 (Thermo Fishers Scientific, Waltham, MA, USA) for 30 min and then mounted with ProLong Gold antifade reagent with DAPI (Thermo Fisher Scientific, Waltham, MA, USA) after formalin fixation. Images were acquired using a confocal laser scanning microscope (CLSM) LSM-700 (Carl Zeiss MicroImaging GmbH, Jena, Germany). 

### 4.4. Seahorse XF-24 Analysis in Hepatocytes

Cellular respiration was assessed using an XF24 analyzer (Seahorse Bioscience, Billerica, MA, USA) as previously described [38]. AML12 hepatocytes, at a density of 1 × 10^4^ cells per well in a XF24 seahorse plate, were co-treated with 100 μM BSA-conjugated PA (Sigma-Aldrich, St. Louis, MO, USA) and/or GPA for 24 h. The oxygen consumption rate was measured in XF assay media (25 mM glucose, 1 mM sodium pyruvate in XF basal medium) for cellular respiration analysis. All the reagents were purchased from Sigma-Aldrich (St. Louis, MO, USA) and diluted in XF assay media (Seahorse Bioscience, Billerica, MA, USA) and loaded into ports of the flux plate (1 μg/mL oligomycin, 1 μM carbonyl cyanide-p-trifluoromethoxyphenylhydrazone [FCCP]), and 2 μM antimycin A in sequence as the final working concentrations). 

### 4.5. Adipogenesis and Bodipy^TM^ Staining in Adipocytes

First, 3T3-L1, a murine preadipocyte cell line, was cultured at 37 °C under 5% CO_2_ in DMEM with 100 U/mL of penicillin, 100 μg/mL streptomycin and 10% (*v*/*v*) heat-inactivated calf serum (Thermo Fisher Scientific, Waltham, MA, USA). For differentiation, 3T3-L1 cells, at a density of 1.5 × 10^4^ cells per well in a 24-well plate, were treated with DMEM containing 10% FBS, 100 U/mL of penicillin, 100 μg/mL streptomycin, 0.5 mM IBMX, 1 μM dexamethasone and 10 μg/mL insulin solution for 2 d. Subsequently, the cells were refreshed every 2 d with medium devoid of IBMX and dexamethasone for 7 d. The treated cells were stained with Bodipy^TM^ 493/503 and mounted with ProLong Gold Antifade Reagent and DAPI after formalin fixation. Images were acquired using a fluorescence microscope (Nikon ESCLIPSE Ts2; Nikon Corporation, Tokyo, Japan). For the measurement of the fluorescence intensity, Bodipy^TM^-stained lipids in the adipocyte were eluted with 5% Nonidet™ P-40 (Sigma-Aldrich, St. Louis, MO, USA) buffer and were measured at an excitation wavelength of 480/30 nm and an emission wavelength of 530/30 nm using a VICTOR Nivo™ Multimode Microplate Reader (PerkinElmer, Waltham, MA, USA).

### 4.6. Animal Study

Male C57BL/6N mice (10-week-old for CDA and 8-week-old for HFD) were purchased from Orient Bio Inc. (Seongnam, Republic of Korea) and were housed in a specific pathogen-free facility under controlled temperature (22 ± 1 °C), humidity (55 ± 10%), and lighting (12 h light/dark). The mice had free access to water and were provided ad libitum with one of the following diets: L-amino acid diet with 60 kcal% fat, 0.1% methionine, and no added choline (CDA, A06071302, Research Diets, New Brunswick, NJ, USA), 60% HFD (D12492, Research Diets), or regular chow diet (5053, LabDiet, St. Louis, MO, USA). After a one-week acclimation period, the animals were fed CDA for 3 weeks or HFD for 4 weeks. GPA was intraperitoneally injected daily at a concentration of 10 mg per kg of body weight during the final week. The body weight and food intake were monitored weekly for all the mice, and the body composition was measured using ^1^Hnuclear magnetic resonance (NMR; Bruker Optics, Billerica, MA, USA) before fasting. After overnight fasting, the mice were euthanized following anesthesia with 2% isoflurane gas, and tissues were harvested for further analysis. All the animal experimental procedures were approved by the Institutional Animal Care and Use Committee of Gachon University.

### 4.7. Blood Parameters

After anesthesia, blood samples were collected via cardiac puncture from overnight-fasted mice, and plasma was obtained by means of centrifugation for 20 min at 3000× *g*. The AST and ALT levels were measured in the plasma using a Cobas c111 analyzer (Roche Diagnostics, Rotkreuz, Switzerland). The plasma TG was measured using L-Type Triglyceride M (Fujifilm Wako Pure Chemical Corporation, Osaka, Japan) according to the manufacturer’s instructions. 

### 4.8. Histopathology and NASH Activity Scoring

Preparation and staining of the histopathological samples were performed at the core facility of the Institutional Animal Care and Use Committee at Gachon University. Briefly, formalin-fixed liver samples were washed with tap water, dehydrated in an alcohol-xylene series, and embedded in paraffin. Paraffin sections were stained with hematoxylin and eosin (H&E) or Sirius red for the histopathological NASH activity scoring, as previously described with slight modifications [39,40]. Briefly, five randomly selected areas were assessed under an optical microscope at × magnification of ×200, and scored for steatosis (0–3), inflammation (0–3), ballooning (0–2), and fibrosis (0–4). The average score for each individual was calculated and the NAS value was determined by summing the scores for each individual.

### 4.9. RNA Sequencing and Gene Set Enrichment Analysis (GSEA)

RNA was extracted from 3T3-L1 cells using a RNeasy Mini Kit (QIAGEN, Hilden, Germany). The RNA quality was assessed using an Agilent 2100 Bioanalyzer with an RNA 6000 Nano Chip (Agilent Technologies, Amstelveen, The Netherlands). The library construction was performed using a QuantSeq 3′-mRNA-Seq Library Prep Kit (Lexogen, Vienna, Austria) according to the manufacturer’s instructions at the Genomic Medicine Institute Research Service Center and sequenced on the Hiseq platform (Illumina, San Diego, CA, USA) at 2 × 150 bp read length. Raw data quality control was performed using FastQC (v0.11.9). Each read’s unique molecular index (UMI) was extracted with the “extract” function from UMI tools (1.1.2). Trimming was performed using the bbduk.sh script from BBMap software (38.87), providing Illumina sequencing adapters and polyA fasta as reference files. The reads were then mapped to a mouse reference (GRCm38/mm10) using the STAR aligner (2.7.9a) together with Samtools (1.13+htslib-1.13). The PCR duplicates were removed using the UMItools dedup in Python 3.8.12. For the transcript quantification, htseq was used to detect the gene counts.

GSEA was conducted using the Java GSEA desktop application (GSEA v4.2.1) [41]. Pre-ranked options were employed based on the fold changes in the GPA-treated cells compared with the vehicle-treated cells, and hallmark gene sets were used. Given the limited sample size in this study, *p*-values were calculated by permuting the data 1000-fold to identify enriched gene sets. Statistical significance for the gene sets was set at *p* < 0.05.

### 4.10. Real-Time Quantitative (q)PCR

The total RNA was extracted from the cell lines and snap-frozen tissues of overnight-fasted animals using TRIzol reagent (Thermo Fisher Scientific, Waltham, MA, USA). The RNA was quantified at 260/280 nm using a NanoDrop 2000C spectrophotometer (Thermo Fisher Scientific, Waltham, MA, USA). The RNA was reverse transcribed using a TOPscript TM RT DryMIX kit according to the manufacturer’s protocol (Enzynomics, Daejeon, Republic of Korea). Real-time PCR was performed using the Applied Biosystems 7300 Real-Time PCR System (Thermo Fisher Scientific, Waltham, MA, USA). The primer sequences used in this study are listed in Supplementary Table S4.

### 4.11. Statistics

All the data were expressed as the mean ± standard error of the mean (SEM). The significance of the differences between two groups was evaluated using a two-tailed unpaired Student’s *t*-test and the significant differences were expressed as sharp “^#^” as follows: “^#^”, *p* < 0.05; “^##^”, *p* < 0.01. More than three groups were evaluated using a one-way or two-way analysis of variance (ANOVA) followed by Bonferroni’s post hoc analysis and were expressed as asterisks “*” as follows: “*”, *p* < 0.05; “**”, *p* < 0.01; and “***”, *p* < 0.001 using GraphPad Prism software (ver. 5.0.1). Significance was denoted for all the groups exhibiting statistical significance.

## Data Availability

All data generated or analysed during this study are included in this published article and its supplementary information files.

## Supplementary Materials

The following supporting information can be downloaded from https://www.mdpi.com/article/10.3390/ijms25021096/s1.
