# Supplementary material for: Graphislactone A, a Fungal Antioxidant Metabolite, Reduces Lipogenesis and Protects against Diet-Induced Hepatic Steatosis in Mice
Source: Int J Mol Sci. 2024 Jan 16;25(2):1096. doi: 10.3390/ijms25021096 (PMC10816634; doi:10.3390/ijms25021096)
Supplement: Supplementary file 1 [file ijms-25-01096-s001.zip › Supplementary Figure_final.pptx]

## Slide 1
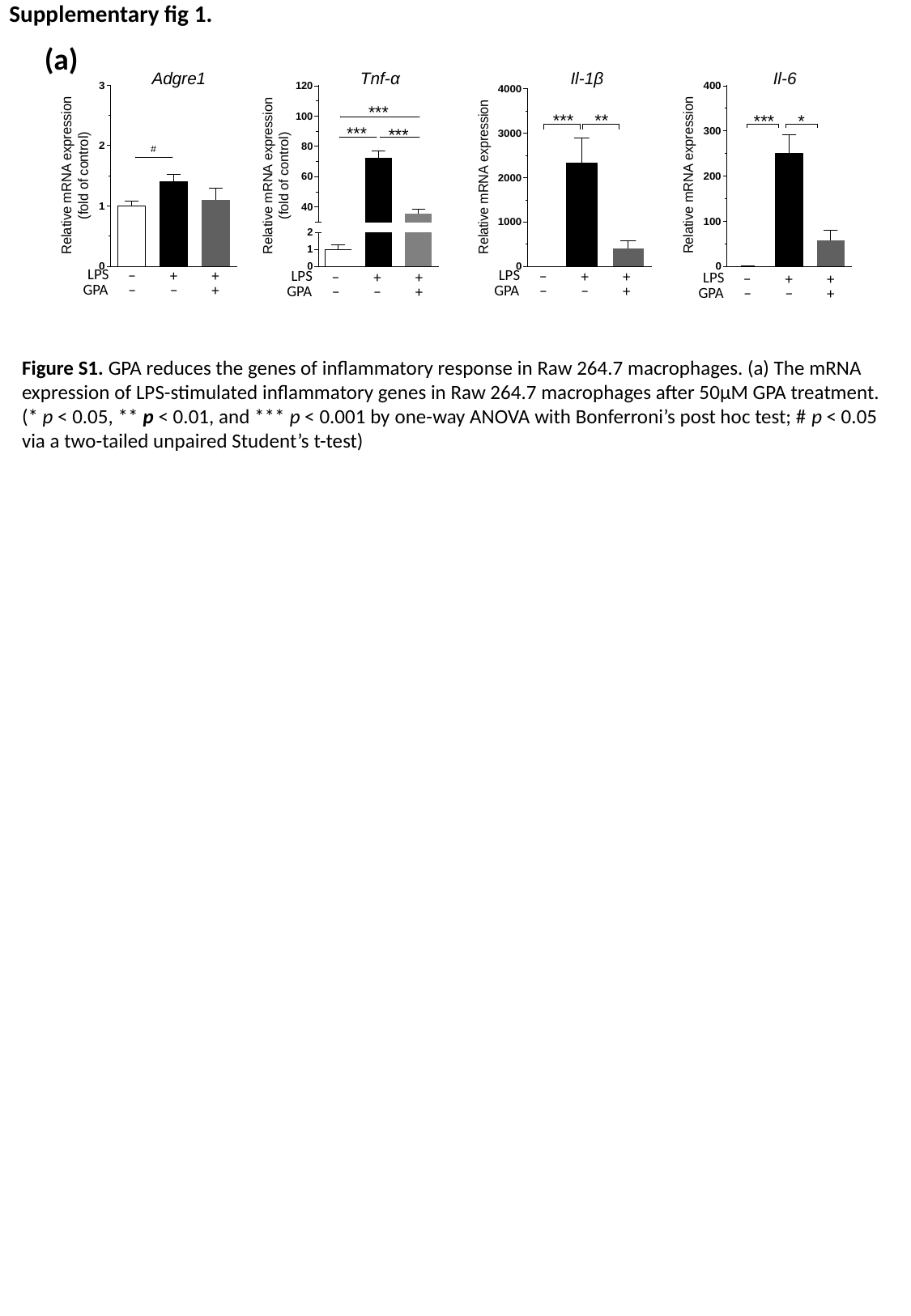

Supplementary fig 1.
(a)
Tnf-α
Adgre1
Il-1β
Il-6
LPS
−
+
+
GPA
−
−
+
LPS
−
+
+
GPA
−
−
+
LPS
−
+
+
GPA
−
−
+
LPS
−
+
+
GPA
−
−
+
Figure S1. GPA reduces the genes of inflammatory response in Raw 264.7 macrophages. (a) The mRNA expression of LPS-stimulated inflammatory genes in Raw 264.7 macrophages after 50μM GPA treatment. (* p < 0.05, ** p < 0.01, and *** p < 0.001 by one-way ANOVA with Bonferroni’s post hoc test; # p < 0.05 via a two-tailed unpaired Student’s t-test)
