# Supplementary material for: Graphislactone A, a Fungal Antioxidant Metabolite, Reduces Lipogenesis and Protects against Diet-Induced Hepatic Steatosis in Mice
Source: Int J Mol Sci. 2024 Jan 16;25(2):1096. doi: 10.3390/ijms25021096 (PMC10816634; doi:10.3390/ijms25021096)
Supplement: Supplementary file 1 [file ijms-25-01096-s001.zip › Supplementary Table S4-R1 (1).pdf]

**Supplementary Table S4. The primer sequences used in Quantitative RT-PCR analyses**

| Gene | Forward Primer Sequence (5' ->3') | Reverse Primer Sequence (5' ->3') |
|---|---|---|
| Mouse *Adgre1* | TCATCAGCCATGTGGGTACAG | CACAGCAGGAAGGTGGCTATG |
| Mouse *Tnf-α* | CCAGACCCTCACACTCAGATC | CACTTGGTGGTTTGCTACGAC |
| Mouse *Il-1β* | AGGCAGGCAGTATCACTCATTGT | GGAAGGTCCACGGGAAAGA |
| Mouse *Il-6* | GCTACCAAACTGGATATAATCAGGA | CCAGGTAGCTATGGTACTCCAGAA |
| Mouse *Pparγ* | ATCTTAACTGCCGGATCCAC | TGGTGATTTGTCCGTTGTCT |
| Mouse *Pparα* | AACTGGATGACAGTGACATTTCC | CCCTCCTGCAACTTCTCAAT |
| Mouse *CPT1α* | TGGGCTACTCAGAGGATGG | AAGGTGTCAAATGGGAAGG |
| Mouse *Acsl1* | TGCCAGAGCTGATTGACATTC | GGCATACCAGAAGGTGGTGAG |
| Mouse *Dgat2* | GCGCTACTTCCGAGACTACTT | GGGCCTTATGCCAGGAAACT |
| Mouse *Acox1* | TAACTTCCTCACTCGAAGCCA | AGTTCCATGACCCATCTCTGTC |
